# Hyperglycemia as a Risk Factor of Ischemic Stroke

**DOI:** 10.4172/2157-7609.1000153

**Published:** 2013-06-29

**Authors:** Ziyan Zhang, Jingqi Yan, Honglian Shi

**Affiliations:** Department of Pharmacology & Toxicology, University of Kansas, Lawrence, Kansas, USA

**Keywords:** Diabetes, Stroke, Hyperglycemia, BBB, Neuroprotection

## Abstract

Diabetes is considered a major risk factor for stroke and is associated with worsened stroke outcomes. Here, we discuss and summarize the mechanisms that have been associated with the increased risk of stroke due to the hyperglycemia in diabetes mellitus. In diabetic stroke models, hyperglycemia exaggerates the following damaging processes: acidosis, accumulation of reactive oxygen species/reactive nitrogen, inflammation and mitochondrial dysfunction. Understanding the mechanism of diabetes acting as a stroke risk factor will definitely assist to reveal issues related to drug metabolism and toxicity in diabetic stroke. In addition, it is suggested that future studies may focus on the mechanisms mediating blood-brain barrier and astrocytes dysfunction under hyperglycemic stroke.

## Introduction

According to the World Health Organization, over 15 million people, equating to one in every 400 people, suffer a stroke worldwide a year [1]. Stroke is a leading cause of mortality after heart disease, and is responsible for about 9% of total deaths each year. It is also the single most common cause of long-term disability, with up to 40% of stroke patients not expected to recover independence [2]. A stroke can be due to ischemia caused by thrombosis or embolism or due to a hemorrhage. Ischemic stroke accounts for approximately 80–85% of all cases and is characterized by the disruption of cerebral blood flow and lack of oxygen to the affected area [3].

There is a substantial amount of clinical and experimental data showing the relationship between diabetes and stroke [4–6]. Diabetes is considered a risk factor particularly for ischemic stroke. Patients with diabetes are at 1.5–3 times the risk of stroke compared with the general population [7]. Diabetes also doubles the risk of stroke recurrence [8]. Furthermore, stroke outcomes are significantly worse among diabetic patients with increased mortality and neurological and functional disabilities [9]. Diabetes is a complex disease that extends beyond dysfunctional glucose regulation. People with diabetes generally have additional stroke risk factors, including hypertension, dyslipidemia, obesity, and atrial fibrillation. This review aims to discuss and summarize the mechanisms that have been associated with the increased risk of stroke due to the hyperglycemia in diabetes mellitus. According to a variety of findings in the literature, four main pathways, including acidosis, reactive oxygen species/reactive nitrogen species, inflammation, and mitochondrial dysfunction are involved in hyperglycemia-aggravated stroke.

## Acidosis

Acidosis is considered to be a major contributor to neuronal damage in cerebral ischemia. Glucose is the sole energy substrate in the brain during both aerobic and anaerobic conditions. During anaerobic conditions such as in cerebral ischemia, glycolysis is the only process capable of producing significant amounts of ATP and lactate is the main product of glycolysis [10]. Hyperglycemia can worsen ischemic outcomes through aggravating acidosis in ischemic brain tissues. In 1977, Myers and Yamaguchi first reported that glucose administrated before cerebral ischemia significantly exacerbated the post-ischemic outcome [11]. Siesjo’s lab conducted a series of experiments and reached the conclusion that infusion with glucose pre-ischemia led to the excessive amount of lactic acid. The accumulation of lactate resulted in a decrease of pH which is responsible for the excess damage in brains [12]. The exacerbation of post-ischemic brain injury is associated with enhancement of excitatory amino acid release, toxic metabolism of NO, and hydroxyl radical formation due to the ischemia-acidosis [13].

Furthermore, neuroprotection provided by inhibiting glycolysis supports that acidosis plays important role in brain injury caused by ischemia and hyperglycemia. For example, pretreatment with 2-Deoxy-D-glucose (2DG), which can inhibit glycolysis, reduced mortality and morbidity in hyperglycemic rats under the condition of four-vessel occlusion [14]. 2DG also exerted a cytoprotective effect by preventing ischemia-induced hippocampal neuron damage in a gerbil transient forebrain ischemia model. By using ^1^H NMR and MRI, Wei et al. concluded that inhibition of glucose metabolism by 2DG had a beneficial effect in reducing brain injury and minimizing the lactate production in brain during middle cerebral artery occlusion (MCAO)-reperfusion in hyperglycemia rats [15].

However, it is still arguable whether lactate accumulation is directly detrimental to the ischemia brain. It has been reported that in an *in vitro* ischemia model, the combination of high glucose and acidosis, but not acidosis per se or the combination of lactate and acidosis exacerbated damage [16]. On the other hand, increased glucose concentrations also exacerbated ischemic injury in brain slices even when pH was tightly controlled [17]. Moreover, there is evidence demonstrating that pretreatment with high glucose (20mM) before hypoxia and mild acidosis has some protective effects against ischemia [10].

## Reactive Oxygen Species (ROS)

During stroke, excessive production of ROS can lead to breakdown of the BBB and focal lesions. ROS is a group of natural by-products of oxygen metabolism including hydroxyl radical, superoxide and hydrogen peroxide. ROS have many detrimental effects, such as lipid peroxidation, protein denaturation, inactivation of enzymes, nucleic acid and DNA damage, release of Ca^2+^ from intracellular store, damage to the cytoskeletal structure and chemotaxis. All of these can lead to cell death and tissue destruction [18].

Several studies have demonstrated that hyperglycemia enhances the generation of ROSduring ischemia-reperfusion process [19,20]. Superoxide anion radical (O_2_^−․^), the primary ROS, has been implicated in four major pathways leading to hyperglycemic complications: glucose-induced activation of protein kinase C (PKC) isoforms, increased formation of advanced glycation endproducts, increased glucose metabolism by aldose reductase pathway, and increased flux through the hexosamine pathway. Increased glycolysis results in higher NADH production and increased electron transfer through the electron transport chain in the mitochondrion. This results in the accumulation of a number of intermediates, which can transfer an electron to molecular oxygen to form O_2_^−․^ In addition, PKC-dependent activation of NADPH oxidase is also a significant source of O_2_^−․^ in diabetes [21].

In diabetic rats, interestingly, MCAO for 2h increased NADPH oxidase subunit expression to much higher levels than ischemia-reperfusion (I/R) alone [22]. It was reported that activation of Rac and subsequently of the gp91 phox containing NADPH oxidase promoted cerebral ROS formation, which led to disruption of blood brain barrier [23]. Indeed, inhibition of NADPH oxidase was neuroprotective after I/R [24]. It was also reported that post-ischemic O_2_^−․^ production and cell death were prevented or reduced by decreased glucose metabolism or inactivation of NADPH oxidase both *in vitro* and *in vivo*, identifying glucose as a requisite electron donor for reperfusion-induced neuronal O_2_^−․^ production [25]. By using a novel electrochemical O_2_^−․^ sensor, Tsuruta et al. demonstrated that following reperfusion from forebrain ischemia, hyperglycemia enhanced superoxide generation and lipid peroxidation in the brain [26].

ROS might play a central role in blood brain barrier (BBB) dysfunction during ischemia-reperfusion. They can change the vascular tone and therefore influence cerebral blood flow. Their vascular effects also include increasing platelet aggregability and endothelial cell permeability, altering reactivity to vasodilators, and leading to the formation of focal lesions in endothelial cell membranes [18]. Kamada et al. reported that hyperglycemia increased oxidative stress and matrix metalloproteinases-9 (MMP-9) activity, exacerbating BBB dysfunction after I/R [27]. Other studies showed that ROS induced degradation of the basement membrane and enhanced tyrosine phosphorylation of tight junctions by activating MMP-1,2,9 and decreasing tissue inhibitor of MMP (TIMP-1 and 2). Therefore, ROS production led to increased permeability and monocyte infiltration [28].

## Reactive Nitrogen Species (RNS)

Reactive nitrogen species has also been reported to contribute to the exacerbated damage in stroke with diabetes [29,30]. Two important reactive nitrogen species are nitric oxide (NO) and peroxynitrite (ONOO^−^). NO first known as endothelium-derived relaxing factor, which is induced during ischemia to increase vascular blood perfusion through increased expression of nitric oxide synthase (NOS) [31,32]. Since ischemia also increases the production of superoxide, the excessive nitric oxide forms a large amount of peroxynitrite [30]. Peroxynitrite induces damage in brain cells by directly causing protein S-nitrosylation, DNA fragmentation, and lipid peroxidation [33]. Moreover, peroxynitrite disrupts blood-brain barrier structure and increases the permeability [34]. Excessive peroxynitrite is also formed in diabetic vasculature and plays an important role in diabetes-induced vascular damage, both in experimental models and in humans [35,36]. High glucose levels result in increased nitric oxide and superoxide production, leading to peroxynitrite formation [37–39]. Given both ischemia and hyperglycemia induces the formation of peroxynitrite, the peroxynitrite level in diabetic stroke was much higher than non-diabetic stroke [30]. The immunoreactivity of nitrotyrosine, products of proteins oxidation by peroxynitrite, is more prominent in hyperglycemic stroke [29]. Reducing the peroxynitrite formation in hyperglycemic MCAO rat brains with NOS inhibitor, L-nitroarginine methyl ester (L-NAME), could decrease the infracted region to the levels observed in normoglycemic rats [13]. In this way, peroxynitrite may serve as one of the mediators for the severe brain damage in diabetic stroke.

## Inflammation

Accumulating evidence suggests that cerebral ischemia elicits inflammation. During ischemia, the circulating cells including neutrophils, monocytes/macrophages and resident cells including microglia, astrocytes, and endothelial cells secrete inflammatory cytokines in the damaged areas [40]. Inflammatory cytokines such as tumor necrosis factor-alpha (TNFα) and interleukin-1beta (IL-1β) cause cellular adhesion molecule expression on endothelial cells which increases polymorphonuclear (PMN) leukocytes and other inflammatory cells to adhere to the endothelial cells [41]. These cell-bound leukocytes then release MMPs, which participate in the breakdown of neurovascular matrix with consequent BBB disruption and edema. Furthermore, adhesion of leukocytes induces burst of production of ROS that contribute to secondary injury of BBB [42].

Many studies have provided evidence for a linkage between diabetes and inflammation. Nuclear factor kappa B (NF-κB) controls the induction of many inflammatory genes. During hyperglycemia, NF-κB is rapidly and dramatically activated in vascular cells and results in a subsequent increase in leukocytes adhesion and transcription of pro-inflammatory cytokines [6]. Glucose intake also causes an increase in early growth response-1 (Egr-1) that modulates the transcription of tissue factor (TF). Aljada et al. reported that glucose-induced increase in TF worsened ischemic damage by promoting coagulation in local capillaries [43]. Kim et al. found that chronic high glucose exposure of leukocytes increased their binding to human aortic endothelial cells [44]. Panes et al. observed diabetic hyperglycemia was associated with exaggerated leukocytes-endothelial cell adhesion and albumin leakage in response to I/R, setting a stage for an increased inflammatory response [45].

There are several reports suggesting that inflammatory responses might mediate hyperglycemia-aggravated brain damage induce by I/R. At post-translational levels, IL-1β and COX-2 expressions were significantly higher following hyperglycemic ischemia than hyperglycemic shams [46]. By using myeloperoxidase immunohistochemistry, Lin et al. demonstrated that hyperglycemia triggered early, massive deposition of neutrophils in the post-ischemic brain, which might exacerbate injury [47]. This increased adhesion of leukocytes to the endothelial cells was largely due to the increased expression of cell adhesion molecules on endothelial cells. It was reported that the number of intercellular adhesion molecule 1 (ICAM-1)-stained microvessels in the cortex was markedly increased at 3 days following I/R in diabetic, but not non-diabetic rats. Western blott showed that IL-1β was increased after 3 days of reperfusion in diabetic rats, suggesting that IL-1β might mediate ICAM-1 expression in diabetic animals [48]. Tsuruta et al. demonstrated that hyperglycemia enhanced the expression of high-mobility group box 1 (HMGB1) in the brain cytoplasm. HMGB1 is reported to be released early from neurons following ischemic injury. It acts as an upstream inflammatory signal by inducing other pro-inflammatory cytokines expression [26]. Therefore, the elevation of HMGB1 during hyperglycemic ischemia may contribute to inflammatory response.

## Mitochondrial Dysfunction

Another mechanism involving worsened outcomes of stroke is diabetes-induced mitochondrial dysfunction. Moreira et al. demonstrated that mitochondria isolated from the brains of STZ-induced diabetic rats possessed a lower content of antioxidant defenses (CoQ) and higher oxidative stress than control [49]. The activities of mitochondrial enzymes, NADH dehydrogenase, succinate dehydrogenase and cytochrome oxidase, were decreased in diabetic rat brains [50]. Increased cytochrome C and active caspase-3 levels were also observed in cytosol of diabetic rat brain cells [50]. The impaired mitochondrial functions by diabetes make the neurons more vulnerable to stroke because ischemia itself also induces mitochondrial dysfunction through oxidative stress and glutamate release [51,52], increases mitochondrial ROS production, reduces respiratory complex activities, and causes the release of cytochrome C, all of which serve as important pathways for stroke-induced neuron death [53,54]. It has been reported that severe neuron death in diabetic rats after MCAO was associated with mitochondrial dysfunction and increased cytochrome C release [55].

Besides neuronal cells, diabetes was reported to impair mitochondrial functions in brain endothelial cells [56]. In diabetes, endothelial cells apoptosis and dysfunction was induced by increased mitochondrial ROS generation, impaired mitochondrial energy production, and release of apoptotic factors [57–60]. It was suggested that vasculatures needed to respond quickly to increased blood perfusion and attenuate the inflammatory reactions to protect brain from ischemic damage [61]. Normally, endothelial cells secrete vasodilator molecules to modulate vascular tone and increase blood supply during ischemia [61,62]. Nitric oxide, one of the endothelial vasodilators, can suppress inflammatory reactions by preventing leukocyte adhesion and reducing the expression of pro-inflammatory factors [63]. Therefore, both endothelial cell death and dysfunction limit their capacity of responding to ischemic damage. However, more research is needed to understand the mechanisms of diabetes-induced mitochondrial dysfunction-mediated vascular dysfunction and exacerbated outcomes of stroke.

## Other Related Factors

There are other mediators which are not tightly related to the above mechanisms but regulate brain damage during hyperglycemic ischemia. Hypoxia inducible faction 1 (HIF-1) is a key factor in mediating a series of genes that induce erythropoiesis, apoptosis, anti-apoptosis, necrosis, and angiogenesis during ischemic brain injury. In tissues from diabetic animals and patients, HIF-1α functional activity decreased due to impaired HIF-1α binding to the coactivator p300. In setting of hypoxia, vascular endothelial cell growth factor (VEGF) is directly controlled by HIF-1. VEGF production in response to hypoxia was decreased in diabetic tissues [64]. In acute hyperglycemia-induced hemorrhagic transformation in a rat model of focal cerebral ischemia, the inhibition of HIF-1α and its downstream genes attenuated hemorrhagic transformation (extravasation of blood cellular elements), reduced cerebral infarction and ameliorated neurological deficits [65].

Rho and Rho-associated kinase (ROCK) play pivotal roles in the pathogenesis in stroke. ROCK activation is considered to increase the risk of cerebral ischemia and worsen the ischemic tissue outcome and functional recovery. Rho/ROCK activity was increased systemically as well as in cerebral arteries in both Type 1 and Type 2 diabetic animal models [66]. As a result, Rho is considered to be involved in the increased risk of stroke in diabetes.

## Future Direction

Many studies suggest that hyperglycemia worsens the outcome and increases the risk of ischemia. The process is complex. The possible mechanisms are interdependent, interactive, and possibly modulate each other. However, it seems that ROS play a central role in all the possible mechanisms modulating increased brain damage. For example, acidosis can enhance formation of free radicals. Hyperglycemia-induced oxidative stress and generation of ROS is also responsible for the activation of many inflammatory cytokines while the subsequent inflammation response will in turn generate large amount of ROS.

Though many studies focused on diabetes-induced vascular complications, there are few studies specifically focused on the mechanisms of BBB dysfunction under hyperglycemic stroke. Further research can be focused on the mechanism of BBB’s damage.

It is known that during the development of diabetes a number of biochemical and mechanical factors converge on the endothelium, resulting in endothelial dysfunction and vascular inflammation. This provides a basis for the vascular disease seen in diabetes [67]. It is also reported that during inflammation, HIF-1 levels were upregulated in neutrophils and macrophage [68]. However, there is no research on HIF-1’s effects on cerebral vascular inflammation in diabetes. Besides, HIF-1 plays an important role in modulating BBB permeability during stroke. The increasing permeability of BBB in stroke can provide more oxygen and nutrients to neuronal cells, however, it can also cause brain edema due to leakage of blood components [69]. We postulate that diabetic inflammation may regulate activity of HIF-1 in brain endothelial cells, leading to aggravation of brain injury in stroke.

Astrocytes are glial cells that envelop >99% of the BBB endothelium. Interaction of astrocytes with endothelial cells greatly enhances endothelial cell tight junctions and maintains BBB tightness and function [70]. Hypoxic exposure induced a higher level of VEGF releasing from astrocytes, which provides protective effect to neighboring cells [71]. High glucose can potentiate the increase of hemichannel activity and impairment of gap junctional communication among astrocytes under hypoxia. Thus, hyperglycemia resulted in astrocytes dysfunction and death [72]. It is highly possible that in hyperglycemic stroke, dysfunction of astrocytes is a major factor resulting in BBB damage.

So far numerous neuroprotective drugs have been investigated in animal models of ischemic stroke, many of which have achieved general success in preclinical studies across disparate animal models [73]. However, of the more than 100 neuroprotective agents that reached randomized clinical trials in focal ischemic stroke, none has proven unequivocally efficacious, despite success seen in preceding animal studies [74]. The translational disappointment of neuroprotective agents likely arise from a combination of factors including poor choice of the agents and time of administration, the molecular mechanism targeted, and difference between animal models and human pathological conditions [75].

**Scheme 1:**
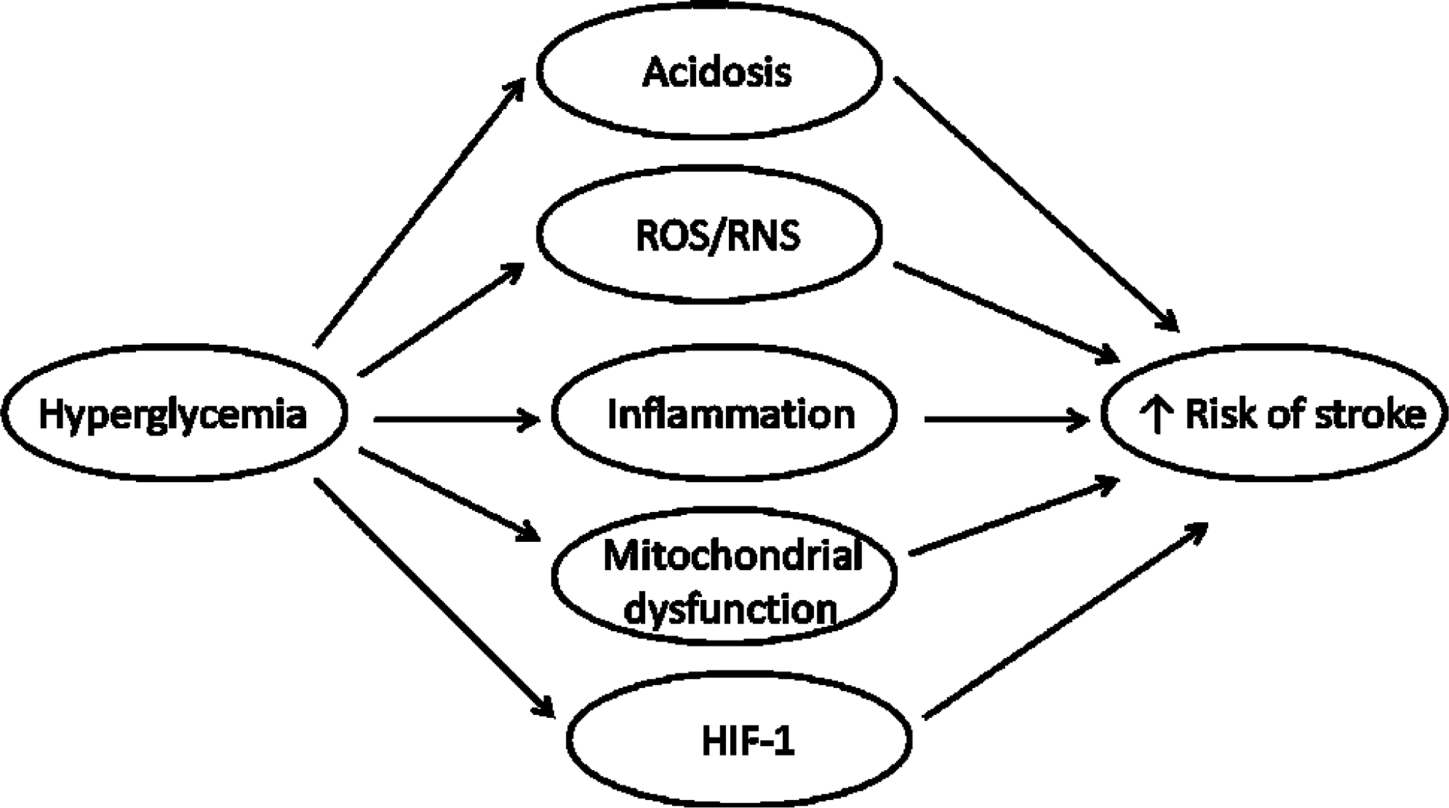
Possible mechanism of hyperglycemia-mediated increased stroke occurrence and exacerbated stroke outcome.

In summary, hyperglycemia may increase stroke occurrence and exacerbate stroke outcome through modulating acidosis, free radical generation, mediators of inflammation, mitochondrial function and other factors such as HIF-1 (Scheme 1). All the factors may have impact on drug metabolism and toxicity in diabetic stroke.
